# Intracellular Eukaryotic Pathogens' Virulence Attributes and Their Interplay with Host Immune Defenses

**DOI:** 10.1155/2017/1264974

**Published:** 2017-11-05

**Authors:** Anamélia Lorenzetti Bocca, Célia Maria de Almeida Soares, Joshua D. Nosanchuk, Ildinete Silva-Pereira

**Affiliations:** ^1^Cell Biology Department of Biology Institute, Laboratory of Applied Immunology, University of Brasília, 70910-900 Brasília, DF, Brazil; ^2^Laboratory of Biochemistry and Molecular Biology of the Interaction between Host-Pathogenic Fungi, Biology Institute, University of Goiás, 74001-970 Goiânia, GO, Brazil; ^3^Departments of Medicine and Microbiology & Immunology, Albert Einstein College of Medicine, Bronx, NY 10461, USA; ^4^Cell Biology Department of Biology Institute, Laboratory of Molecular Biology of Pathogenic Fungi, University of Brasília, 70910-900 Brasília, DF, Brazil

The incidence of life-threatening infections by intracellular eukaryotic pathogens has risen sharply as a result of modern medical care that diminishes immunity in patients, such as invasive catheters, chemotherapy, and steroids, as well as the increased incidence of immunosuppressive diseases, such as those due to the human immunodeficiency virus (HIV). For example, *Cryptococcus neoformans* is responsible for between ~180,000 and 600,000 deaths annually, primarily in sub-Saharan Africa and principally in individuals with HIV [1, 2]. Thus, there is an urgent need for new and deeper insights into the pathobiology of these diverse intracellular invaders' mechanisms for virulence including their strategies to evade or subvert host immune defenses.

Host-pathogen interactions are complex, dynamic, and multifactorial processes. In order to survive and proliferate within the host, eukaryotic pathogens must be able to sense different host microenvironment signals and regulate transcription and translation reprogramming resulting in metabolic adaptations, alterations in cellular morphology, and adjustments and remodeling of their surface envelope (cell wall/plasmatic membrane), among other processes. For example, signals derived through the binding of the fungal cell wall by antibody can result in alterations of gene activation [3] or protein loading in released extracellular vesicles [4]. Osmotic changes can lead to dramatic alterations in protein regulation, such as in *Paracoccidioides lutzii* [5]. In this special issue, areas that are discussed include the dynamics of phase variation in response to stressors, regulation of enzyme secretion, and considerations of metabolic routes as drug targets. In this special issue, E. Camacho and G. A. Niño-Vega detail virulence factors that facilitate the survival of *Paracoccidioides* spp. The pathway to the identification and development of new antifungal drugs through studies on antifungal resistance and metabolism is thoroughly addressed in an article by J. A. Parente-Rocha et al.

On the other hand, effective host responses require the ability of the host to recognize and respond to the pathogen employing several mechanisms to eradicate and/or control the pathogen through the activation of an efficient immune response. The host defense mechanisms include harnessing the functions of macrophages, dendritic cells, T cells, B cells, Th1, Th2 & Th17 responses, antibody, and complement as well as the engagement of such cells through recognition receptors such as TLRs, Dectin-1, complement, mannose & other lectin receptors, scavenger receptors, IL-1 receptor, E-cadherein, EGFR-HER2, Gp96, CD14, CD44, and CDw17. For example, dectin-1 is required for the upregulation of miR155 in macrophages challenged with *Candida albicans* [6] and NLRP3 inflammasome activation by *Paracoccidioides brasiliensis* is linked to a protective response against this pathogen [7]. This special edition will examine cellular and humoral systems in responding to intracellular eukaryotic pathogens. Additionally, issues on how vaccination (both with pathogen products or primed cells, such as dendritic cell) can alter the host-pathogen dynamic will be explored. The interplay between the host and pathogen will be highlighted by a focus on the ability of microbes to undergo morphogenesis as a means to escape immune surveillance. For example, the topic of fungal dimorphism and virulence will be carefully detailed at the molecular level by G. M. Gauthier.

Understanding of the interplay between intracellular eukaryotic pathogens and host cells requires dissection at the levels of both pathogen and host. Dynamic ongoing shifts in responses within both the invader cells and the host cells dictate the outcome of the interaction, to the benefit or detriment of each party. The overall complexity of the processes occurring in such struggles is daunting, yet major insights into the pathobiology of these diseases have been achieved. With this special issue, we have provided a platform that presents significant findings that offer insights into host-pathogen interactions.

*Anamélia Lorenzetti Bocca* *Célia Maria de Almeida Soares* *Joshua D. Nosanchuk* *Ildinete Silva-Pereira*
